# The Role of Bezlotoxumab for the Prevention of Recurrent *Clostridioides difficile* Infections: A Review of the Current Literature and Paradigm Shift after 2021

**DOI:** 10.3390/antibiotics11091211

**Published:** 2022-09-07

**Authors:** Melanie L. Hyte, Lee J. Arphai, Charles J. Vaughn, Spencer H. Durham

**Affiliations:** 1 Edward Via College of Osteopathic Medicine, Auburn Campus, Auburn, AL 36832, USA; 2 VA Northeast Ohio Healthcare System, Cleveland, OH 44106, USA; 3 Harrison College of Pharmacy, Auburn University, Auburn, AL 36849, USA

**Keywords:** bezlotoxumab, *Clostridioides difficile*, pharmacotherapy, CDI, recurrence

## Abstract

*Clostridioides difficile* infections (CDIs), and particularly recurrent infections, cause a significant burden on the health-care system. Bezlotoxumab is a new agent for the prevention of recurrent CDIs that has shown strong efficacy and high tolerability in clinical trials. The purpose of this review is to evaluate the published literature for bezlotoxumab, with a focus on literature published since the release of the 2021 focused update to the CDI treatment guidelines. A Medline/PubMed search for “bezlotoxumab” was conducted, resulting in 152 articles. Seventeen studies are included in this review, after excluding non-English-language papers, phase I and II trials, and review articles. Studies published since the 2021 focused update support the recommendations in those guidelines. Furthermore, real-world studies have shown similar results to larger clinical trials. Those with more risk factors for recurrent CDI appear to benefit most from bezlotoxumab. Currently, there are no data to support the use of bezlotoxumab outside current guideline recommendations, but future trials may build on the data seen in real-world studies to further elucidate the place in therapy for bezlotoxumab.

## 1. Introduction

The incidence of *Clostridioides* *difficile* infection (CDI) continues to cause a significant burden to the health-care system, with an estimated annual incidence of over 460,000 infections in the United States alone [1]. The Centers for Disease Control and Prevention (CDC) classifies CDI as an “urgent” threat, the highest level, which means it is associated with serious risks and has the potential to become widespread, and thus requires immediate public health attention [2]. It can significantly affect a patient’s quality of life, leading to missed workdays, impairment during activity, and have prolonged physical and psychological effects, even after infection resolution [3,4]. Additionally, annual recurrences of community-associated and health-care-associated CDI are estimated to be 31,300 and 38,500 cases, respectively [1]. Recurrent CDI (rCDI) cases are becoming particularly problematic, and it has been estimated that between 15% and 35% of patients experiencing a primary CDI episode will have at least one recurrence [5,6,7,8]. The risk of further recurrences increases after each recurrence: after a second episode, the risk of another recurrence increases approximately 40–45%, and after a third episode, the risk increases to more than 60% [9]. In 2017, The Infectious Diseases Society of America (IDSA) and the Society for Healthcare Epidemiology of America (SHEA) released updated guidelines for the treatment of initial and recurrent CDI in adults and children [10]. However, a new agent, bezlotoxumab (BEZ), which is used as an adjunct to standard pharmacotherapy to prevent rCDI in patients, was not available at the time of writing these guidelines. In 2021, the IDSA and SHEA issued a focused update to the 2017 guidelines to address newly published literature, with a major focus on the use of BEZ [11]. Likewise, in 2021, the American College of Gastroenterology (ACG) updated their clinical practice guidelines for the management of CDI and included recommendations regarding BEZ [12]. The ACG guidelines from 2013 did not address BEZ use as it was not available at the time [13]. A comparison of the IDSA/SHEA and ACG guidelines can be seen in Table 1.

Since the publication of these 2021 updated guidelines, additional literature continues to be published regarding the use of BEZ, particularly studies in the real-world setting that support its use to prevent rCDI. Because the IDSA/SHEA focused update provides more in-depth discussion of the clinical trials associated with BEZ, this paper reviews the clinical trials utilized by these guidelines to create their recommendations for BEZ use and focuses on the literature published since the 2021 focused update was released.

## 2. Methods

A Medline/PubMed search was conducted through inception to 20 July 2022 using the term “bezlotoxumab,” which resulted in 152 results. Non-English-language articles and phase I and II clinical trials were excluded. Review articles were also excluded; however, review articles and references in articles were evaluated for relevant data. Four trials were identified that were published prior to the IDSA/SHEA 2021 focused update, but as these were not included in the update, they are not discussed further [14,15,16,17]. Ultimately, 17 studies were included.

## 3. Background

Bezlotoxumab is a novel human IgG1 monoclonal antibody approved by the Food and Drug Administration in October 2016 and is indicated to reduce rCDI in adults ≥18 years of age who are receiving antibacterial treatment for a current CDI and are at high risk of rCDI [18]. High-risk patients include those aged ≥65 years and those who are immunocompromised, have severe CDI, history of prior CDI, or CDI with ribotypes 027/078/244, which are hypervirulent strains associated with an increased production and secretion of toxins that worsens CDI severity and have been associated with poor outcomes [18,19,20]. Toxins A (“enterotoxin”) and B (“cytotoxin”) are thought to be the major virulence factors of *C. difficile*, and infiltrate colonic tissue leading to diarrhea, tissue damage, and inflammation [21,22,23]. Bezlotoxumab provides passive immunity to *C. difficile* toxin B by directly binding to regions of the combined repetitive oligopeptide domains of toxin B, which further obstructs the toxin’s receptor-binding pockets. Once binding has occurred, toxin B is neutralized, preventing toxin B-mediated inflammation and damage to colon cells and subsequent symptoms of CDI [24,25]. Bezlotoxumab does not bind to toxin A and does not disrupt the efficacy of antibacterial therapy used for treatment of the primary CDI. It has no treatment effect on a current CDI episode and should therefore only be administered concurrently with antibacterial therapy for rCDI prevention [18,26].

Bezlotoxumab is administered intravenously (IV) as a single dose synchronously with appropriate antibiotic therapy for CDI. It is available as 1000 mg/40 mL vials with a recommended administration of 10 mg/kg via IV infusion over 60 min [18,27,28]. It requires actual body weight (ABW) dosing since clearance increases proportionally with body weight [29]. The timing of administration does not affect the resolution of CDI, so it can be administered at any point during active antibiotic treatment for CDI [30]. More than a single dose of BEZ or administration after day 14 of active CDI treatment has not been studied and is therefore not recommended [18].

Bezlotoxumab demonstrates primarily intravascular distribution exhibited by a mean volume of distribution of 7.33 L, a half-life of approximately 19 days, and is primarily excreted through catabolism [31]. As a result, drug–drug interactions with BEZ are not expected, and renal or hepatic dose adjustments are not required [32]. No clinically meaningful differences in pharmacokinetics/pharmacodynamics of BEZ have been found based on gender, race, ethnicity, or comorbidities. Use in children and pregnant or lactating participants is not recommended due to a lack of studies in these populations [18].

Bezlotoxumab, like many monoclonal antibodies, has minimal adverse drug reactions (ADRs). The most common ADRs are infusion-related reactions, including nausea, fatigue, pyrexia, dizziness, headache, dyspnea, and hypertension. Common non-infusion-related ADRs include nausea, pyrexia, and headache [18]. Serious ADRs reported within 12 weeks of administration, include sepsis, pneumonia, acute kidney injury, urinary tract infections, and heart failure (HF) [18,19]. Heart failure was more commonly reported in participants who received BEZ compared with placebo. However, the affected patients reported an HF history. A history of HF was further associated with higher mortality rates due to cardiac failure, infection, or respiratory failure in BEZ-treated participants than those who receive placebo. Bezlotoxumab does not have any contraindications, though it should be used with caution in participants with a history of HF only after careful consideration of benefits versus risks. Of note, one patient experienced ventricular tachyarrhythmia approximately 30 min after the start of BEZ infusion, resulting in discontinuation. Finally, while monoclonal antibodies can potentially induce the formation of neutralizing antibodies, this was not seen in clinical trials for BEZ [18].

## 4. Trials Referenced in the 2021 Focused Guideline Update

The 2021 focused update to the CDI clinical practice guidelines notes that patients with a primary CDI and at least one risk factor for a recurrent infection may benefit from BEZ therapy. Logistical concerns, such as cost and requirement of IV therapy, may preclude use in some circumstances. Additionally, patients with multiple risk factors for rCDI and those who have experienced CDI within the prior six months derive the most benefit from BEZ. As such, the guidelines recommend BEZ as a cointervention with standard antibiotics used for treatment in patients currently experiencing a CDI and who have a history of CDI within the last six months [11]. The guidelines utilized several published trials to make this recommendation, and these trials will be examined in the following paragraphs.

The hallmark clinical trials used to bring BEZ to the market are known as MODIFY I and MODIFY II. MODIFY I/II consisted of two independently conducted, randomized, multicenter, double-blind, placebo-controlled trials that occurred from November 2011 to May 2015, representing over 300 sites across 30 countries. The objectives of these trials were to determine the efficacy and safety profile of two full human monoclonal antibodies in preventing rCDI. The 2559 participants from the modified intention-to-treat (mITT) population included adults ≥18 years of age who were prescribed standard-of-care (SoC) antibiotics with metronidazole (MTZ), vancomycin (VAN), or fidaxomicin (FDX) for the treatment of primary rCDI. The participants were randomized 1:1:1:1 to receive a single IV infusion of BEZ only (10 mg/kg), actoxumab only (10 mg/kg; MODIFY I only), BEZ + actoxumab (10 mg/kg each), or placebo, with a recommendation to administer the study medication as soon as possible within the 10–14 days’ treatment with SoC antibiotics. Actoxumab, which targets *C. difficile* toxin A, has been shown to be less effective than BEZ and is not approved for use, and thus outcomes for the actoxumab-only treatment group are not discussed further. Recurrent CDI was defined as a new episode of diarrhea with a positive stool test for *C. difficile* toxins following initial clinical cure (ICC) from baseline episode of CDI and within 12 weeks of receiving infusion of the study medications. The primary end point was the proportion of participants experiencing rCDI following ICC. Rates of rCDI in subpopulation groups were also examined as part of secondary analyses. Rate of sustained clinical cure (SCC), defined as ICC with no CDI recurrence, was a secondary end point, while time to recurrent infection served as an exploratory end point [33].

In the MODIFY I/II trials, 2174 participants (85%) of the mITT population completed the 12-week follow-up period. Forty-seven percent of participants were treated with MTZ, 48% with VAN, and 4% with FDX. More than 90% of the infusions took place within six days of starting SoC. The BEZ treatment group experienced significantly lower rates of rCDI compared to placebo in both trials (MODIFY I: 17% vs. 28%, respectively; adjusted difference [AD], −10.1%; 95% CI, −15.9 to −4.3; *p* < 0.001; MODIFY II: 16% vs. 26%, respectively; AD, −9.9%; 95% CI, −15.5 to −4.3; *p* < 0.001). Bezlotoxumab also demonstrated a significant benefit over placebo in participants receiving oral antibiotic therapy for primary or rCDI without an increased risk of adverse effects. Overall, the number needed to treat (NNT) for BEZ to prevent one rCDI episode was 10 (6 for participants with previous infection and age ≥ 65 years) [33].

Nearly 75% of rCDI developed within the first four weeks following study infusion. Absolute differences in Kaplan–Meier rates for time to rCDI were 12% at weeks 4 and 8 and 13% at week 12 for BEZ versus placebo (*p* < 0.001). Analyses of subpopulations considered to be at high risk of rCDI demonstrated lower rates of rCDI for BEZ only compared to placebo (17% vs. 30%, respectively; AD, −10.0%; 95% CI, −14.0 to −6.0; *p* < 0.0001). The most common ADRs in MODIFY I/II were infusion-related reactions, which were mostly mild/moderate, and almost all resolved within 24 h. Although most ADRs had numerically similar frequencies between the BEZ and placebo groups, no statistical testing was performed. The placebo group had a lower rate of infusion-related reactions than the BEZ-only group (7.6% vs. 10.3%, respectively). Nausea and diarrhea occurred in ≥5% of participants across all study groups with frequencies similar among the BEZ and placebo groups. Infections/infestations were the most common fatal ADRs, occurring in 2% of participants receiving BEZ versus 3% with placebo. Fatal cardiac disorders occurred in 3% of participants who received BEZ compared to 2% in the placebo group [33].

To determine which populations would benefit most from CDI treatment with BEZ, Gerding and colleagues conducted a post hoc analysis of the MODIFY I/II trials to determine the efficacy of BEZ in participants at increased risk of rCDI. Participants were determined based on five characteristics linked with increased risk of rCDI: age ≥65 years old, history of CDI in the previous six months, compromised immunity, severe CDI, and infection with ribotypes 027/078/244. The following end points were analyzed: proportion of participants with ICC, rCDI in 12 weeks, 30-day all-cause and CDI-related hospital admissions, mortality at 30 and 90 days following randomization, and fecal microbiota transplant (FMT) procedures. Of the 1554 participants, 75.6% (*n* = 1175) had at least one pre-specified risk factor for rCDI. Approximately 36% of participants had one risk factor, 27% had two risk factors, and 12% had three or more risk factors. Baseline characteristics were similar when comparing risk-category groups (e.g., BEZ no risk factor vs. placebo no risk factor groups). For participants with one or more risk factors for rCDI, ICC rates were similar between the BEZ and placebo groups (79.6% vs. 80.3%, respectively; difference, −0.7; 95% CI, −5.3 to 3.9). For the five prespecified risk-factor categories, participants in the BEZ group experienced a decreased rate of rCDI compared to placebo, and, notably, all placebo groups within each risk-factor category surpassed a 30% rate in rCDI (Table 2).

CDI recurrence was similar among participants with no risk factors for rCDI. However, in the placebo group, the incidence of rCDI increased with additional numbers of rCDI risk factors. Thus, BEZ appears to reduce the risk of rCDI for participants with one, two, and three or more risk factors compared to participants in the placebo groups (Table 3).

The post hoc analysis demonstrated efficacy of BEZ in reducing rates of rCDI in participants with one or more prespecified risk factors (absolute reduction, −15.9%), with greater decreases associated with three or more risk factors (absolute reduction, −24.8%). Therefore, this study demonstrated that those with at least one prespecified risk factor for rCDI may be most likely to benefit from BEZ treatment [34].

Birch and colleagues conducted a post hoc analysis of the MODIFY trials to determine the impact on clinical outcomes for timing of BEZ administration relative to antimicrobial treatment onset. Timing of study medication administration was categorized as 0–2, 3–4, and ≥5 days following onset of CDI antibiotic treatment. Of the 1554 participants included in the study, 781 and 773 participants received BEZ and placebo, respectively. Baseline characteristics among the two treatment groups at the time of infusion initiation were well-balanced, with a similar 12-week follow-up period completion rate. In the 0–2, 3–4, and ≥5 days after onset of CDI antibiotic treatment groups, 41.8% (*n* = 649), 30.1% (*n* = 469), and 28.1% (*n* = 436) of participants received a BEZ infusion for CDI treatment, respectively. ICC rates were similar for both treatment groups regardless of timing of administration (timing, BEZ vs. placebo: 0–2 days, 81.4% vs. 81.0%; 3–4 days, 77.8% vs. 81.7%; ≥5 days, 80.4% vs. 77.8%). Among all timing categories, the BEZ group demonstrated lower rates of rCDI than the placebo group (timing, rate of rCDI for BEZ vs. placebo, adjusted rate differences: 0–2 days, 19.3% vs. 31.7%, −13.1; 3–4 days, 20.4% vs. 33.0%, −12.3; ≥5 days, 22.8% vs. 35.8%, −12.3). Therefore, the timing of the BEZ administration did not affect the efficacy. Of note, diarrhea in both the BEZ and placebo groups had similar times to resolution, and diarrhea was resolved in nearly 7 out of 10 participants with greater than 2 days of SoC antibiotic treatment prior to infusion [35].

The final two trials referenced in the 2021 focused guideline update were real-world studies related to BEZ. Oksi and colleagues conducted an observational study to examine the efficacy and safety of BEZ for prevention of rCDI. This was a retrospective case-series study conducted at five university hospitals in Finland between April and December of 2017. Forty-six participants who received BEZ and were at risk of rCDI were included in the analysis. Patient risk factors for rCDI included age >65 years, compromised immunity, severe CDI, prior CDI episode(s), infection with hypervirulent ribotypes, hospitalization, inflammatory bowel disease, renal or hepatic impairments, antibiotic use during SoC therapy, antibiotic use after SoC therapy at three months, and use of proton-pump inhibitors. Of note, 78% (*n* = 46) of participants had three or more risk factors for rCDI. Two participants were excluded in the results because of mortality within three months of BEZ administration. One patient had end-stage coronary artery disease and HF and died five days after the infusion, but the physician determined it was a natural event due to comorbidities and not due to the medication. The other patient died from graft-versus-host disease 1.5 months after receiving BEZ. Of the 44 participants, 32 (73%) had not experienced rCDI at three months after receiving BEZ. One patient received BEZ twice and developed recurrent CDI after the second administration. Two participants had infusion-related ADRs: one patient experienced startling sensations after the infusion, and one patient experienced a fever the day after receiving BEZ. Overall, this study provides real-world data and supports the use of BEZ as adjunctive therapy to SoC in the prevention of rCDI [36].

Hengel and colleagues conducted a retrospective, cohort study of 200 participants performed at 34 infusion centers in the United States from April 2017 to December 2018. This study investigated rCDI in participants with primary CDI or rCDI receiving SoC with VAN (68%), FDX (30%), and MTZ (2%). The majority of participants (86%; *n* = 173) had prior CDI episodes before receiving BEZ. Risk factors for rCDI included age ≥65 years, compromised immunity, current CDI episode with severe presentation, and ≥1 CDI episode within the past six months. A total of 158 participants (79%) had ≥2 risk factors. Physicians assessed rCDI ≥90 days after administration of BEZ, defined as recurrence of diarrhea lasting two or more days requiring medical intervention with or without a positive stool test for *C. difficile*. Three participants were lost to follow-up and two participants died within 90 days of receiving BEZ, and as a result, 195 participants were included in the final analysis. Both deceased participants had multiple comorbidities with adverse events that contributed to their death, including a history of HF. Serious ADRs were not observed, no infusion-related reactions were reported in participants after receiving BEZ, and reported ADRs were similar to placebo. Study participants received SoC for a median of 11 days before receiving BEZ. The rates of rCDI were similar in those who received BEZ within seven days of SoC and those who received BEZ after seven days of SoC at 15.2% and 16.2%, respectively. Similar to the Birch study, these findings support that the efficacy of BEZ is not affected by the timing of administration [37].

## 5. Trials Not Included in the 2021 Focused CDI Guideline Update

### 5.1. Studies from the MODIFY Trials

Since the release of the 2021 updated clinical practice guidelines, several studies have been published based on data from MODIFY I/II. Basu and colleagues conducted a post hoc analysis from participants in both trials to assess if BEZ reduced the number of inpatient days compared to placebo over a 12-week period. Inpatient use data was adjusted for survival and censored to estimate cumulative inpatient days for the overall population and subgroups. The primary end point was cumulative inpatient days in any hospital setting during the 84-day follow-up period following receipt of the study infusion. A total of 1554 participants were included in the analysis (781 in the BEZ group, 773 in the placebo group). Participants in the BEZ group spent 2.1 fewer days in the hospital than the placebo group (95% CI, −0.4 to −3.7). These results also held consistent for participants with risk factors for rCDI, including age ≥65 years, compromised immunity, severe CDI, prior CDI, and ribotype 027/078/244 infection. Furthermore, as the number of risk factors increased, treatment with BEZ was associated with greater decreases in inpatient-days compared to placebo (−1.2 days, −2.3 days, −2.5 days, and −3.0 days for zero, one, two, and three or more risk factors, respectively). The greatest reduction in inpatient days (−3.5 days) was observed in participants who presented with severe CDI at study entry. These results show promise for BEZ to reduce inpatient days, particularly for those with multiple risk factors for rCDI [38].

Bouza and colleagues conducted a post hoc analysis to assess BEZ efficacy in the European subgroup of participants enrolled in both the MODIFY I/II trials. The primary end point was rCDI. Secondary outcomes assessed included ICC, SCC, all-cause and CDI-associated rehospitalizations within 30 days of discharge, and mortality through 12 weeks post infusion. A total of 606 of the 1554 participants (39%) enrolled in the MODIFY trials were from Europe, with 313 and 293 in the BEZ and placebo groups, respectively. The primary end point of rCDI was significantly lower in the BEZ group (BEZ 18.2% vs. placebo 29.8%; 95% CI, −11.6, [−19.1 to −4.1]) and for those with one or more risk factors for rCDI (BEZ 19.2% vs. placebo 33.2%; 95% CI, −13.9 [−22.4 to −5.4]). SCC was significantly higher in the BEZ group (BEZ 67.4% vs. placebo 57.0%; 95% CI, 10.4 [2.7 to 18.0]) and for those with one or more risk factors for rCDI (BEZ 65.9% vs. placebo 54.1%; 95% CI, 11.8 [3.1 to 20.2]). ICC was similar between groups (BEZ 82.4% vs. placebo 81.2%: 95% CI, 1.2 [−5.0 to 7.4]). Participants in the BEZ group had lower rates of CDI-related rehospitalization within 30 days of discharge compared to the placebo group (4.9% vs. 14.8%, respectively), and all-cause rehospitalizations and the proportion of participants who died within 30 days or 90 days of randomization were similar between the BEZ and placebo groups (23% vs. 26.6%, 5.1% vs. 5.1%, and 9.9% vs. 10.5%, respectively); however, statistical analyses of these results were not performed [39].

Participants with cancer are known to have a higher incidence of both CDI and rCDI, a lower CDI cure rate, and prolonged time to diarrhea resolution than the general population [40,41,42]. Further, cancer is associated with an increased risk of mortality in hospitalized patients with CDI [43,44]. Cornely and colleagues sought to examine the effects of BEZ in this population by conducting a post hoc analysis of participants with cancer who were enrolled in the MODIFY I/II trials. A total of 382 participants were included: 190 in the BEZ group (75.3% with solid tumors, 27.9% with hematological malignancy) and 192 in the placebo group (76.6% with solid tumors, 28.1% with hematological malignancy). The ICC rates were similar between the BEZ and placebo groups (76.8% vs. 71.9%, respectively; AD, 5.0%; 95% CI [−3.8% to 13.7%]). However, BEZ participants were significantly less likely to experience rCDI than placebo participants (17.8% vs. 30.4%, respectively; AD, −12.6%; 95% CI−22.5% to −2.7%). Based on these results, BEZ appears to be a promising option for cancer participants to prevent rCDI, but further studies are needed to confirm these results [45].

Dubberke and colleagues conducted an analysis of the MODIFY I/II trials to determine if the antibiotic used to treat CDI influenced the BEZ-related decrease in rCDI. In MODIFY I/II, 753 (48.5%) participants received MTZ, 745 (47.9%) received VAN, and 56 (3.6%) received FDX. The mean duration of antibacterial drug treatment prior to the study infusion was comparable among the three drugs (MTZ 3.3 days, VAN 3.2 days, FDX 3.0 days), as was the mean total duration of antibacterial treatment (MTZ 13.6 days, VAN 14.5 days, FDX 11.9 days). A lower number of MTZ recipients experienced a prior CDI episode in the preceding six months (12.9%) or had at least one risk factor for rCDI (66.0%) compared to VAN recipients (41.2% and 83.6%, respectively) and FDX recipients (55.4% and 89.3%, respectively). Rates of rCDI were significantly lower in the MTZ and VAN participants that received BEZ than the ones who received placebo (MTZ: rate difference [RD], –9.7%; 95% CI, –16.4% to –3.1%; VAN: RD, –15.4%; 95% CI, –22.7% to –8.0%). For the FDX group, a statistical difference was not seen between BEZ and placebo (RD, –11.9%; 95% CI, –38.1% to 14.3%), although this may be due to the small number of participants who received the drug. Overall, the results of this analysis suggest that treatment with BEZ is beneficial in decreasing rCDI, regardless of the antibiotic treatment regimen used [46].

Prabhu and colleagues sought to examine 30-day all-cause and CDI-related hospital readmissions for participants in MODIFY I/II who were considered at high risk of rCDI. Only participants who were inpatients at the time of study randomization were included in this post hoc analysis, with a total of 530 participants in the BEZ group and 520 in the placebo group ultimately being included. Patient baseline characteristics were similar between groups. At 30 days after hospital discharge, BEZ participants experienced significantly fewer CDI-related hospital readmissions than placebo participants (absolute difference, −6.1%; 95% CI, −9.5 to −2.8; relative difference, −53.4%). No statistically significant difference was observed between BEZ and placebo related to all-cause hospital readmissions (absolute difference, −3.7%; 95% CI, −9.0 to 1.5; relative difference, −12.1%). Overall, these results show promise for BEZ reducing rCDI-related hospitalizations, but this should be confirmed in larger trials [47].

Lastly, Goldstein and colleagues published a brief report on the extension phase of the MODIFY II trial, which followed some patients beginning at week 12 after BEZ treatment and subsequently followed for nine additional months. A total of 293 participants entered the extension phase who were included in the mITT population of the original study. In the BEZ group, there were no cases of rCDI during the 9-month extension period. The authors concluded that BEZ efficacy was likely due to rCDI prevention as opposed to a delay in rCDI after BEZ concentrations in the body were low. However, the small sample prevents any definitive conclusions [48].

### 5.2. Real-World Trials

Several retrospective studies have been conducted to assess the rate of rCDI in participants who have received SoC monotherapy compared to those who received BEZ with or without SoC [49,50,51,52,53,54]. Table 4 summarizes the results of these studies.

Escudero-Sánchez and colleagues conducted a retrospective, matched-cohort study in Spain using data from two previously published reports [50,55] comparing FDX monotherapy with SoC combined with BEZ. Three hundred and thirty-five participants were evaluated. Participants who received FDX combined with BEZ were excluded (*n* = 13), resulting in a total of 322 participants: 244 in the FDX group and 78 in the SoC plus BEZ group. The FDX group received 200 mg orally twice daily for a mean of 10 days, and the BEZ group received 10 mg/kg IV infusion in addition to SoC, defined as oral VAN (98.7%) for 10 days with or without MTZ. The primary objective was to compare the efficacy in preventing rCDI and the safety of FDX monotherapy versus combination SoC with BEZ in participants with CDI. The study end point was recurrence at 12 weeks. The recurrence rate at 12 weeks was 19.3% in the FDX group and 14.1% in the BEZ cohort (95% CI 0.71- to 2.96; *p* = 0.29). In those participants with no previous episodes of CDI, FDX had a lower recurrence rate (9.4% vs. 15.2%, *p* = 0.07), and BEZ had a lower rate in those with more than one episode (23.5 vs. 35% *p* = 0.35), but neither was statistically significant. Variables associated with recurrence at 12 weeks were used for the multivariate regression model. Results included age >80 years (odds ratio [OR], 1.2; 95% CI, 0.64 to2.39), dementia (OR, 1.7; 95% CI, 0.7 to 0.42), community-onset, healthcare facility-associated (CO-HCFA) episode (OR, 1.8; 95% CI, 1.02 to 3.28), and the presence of previous CDI episodes (OR, 2.0; 95% CI, 0.97 to 3.85). No statistical difference was found in the number of adverse events. The analysis showed both treatments had similar rates of rCDI, regardless of the number of previous episodes [49].

A second trial by Escudero-Sánchez was a retrospective, multicenter, cohort study of 91 participants that received BEZ during the duration of antimicrobial treatment for CDI between July 2018 and July 2019 in 13 Spanish hospitals. The objective of the trial was to verify the efficacy and safety of BEZ in preventing rCDI and to investigate factors related to BEZ failure in a real-world setting. The authors compared the characteristics of study participants to the participants in MODIFY I/II who received BEZ. The following eight variables were examined: age > 65 years; compromised immunity; more than two previous CDIs; severe CDI; infection due to a hypervirulent strain; renal failure; toxin positivity; and treatment with FDX or recurrences. The primary end point was rCDI rate during the 12 weeks after the end of antimicrobial treatment for CDI. The participants generally had a higher risk of rCDI compared to the participants in MODIFY I/II in that they were older (>65 years, 67.0% vs. 49.9%, respectively), had more previous CDI episodes (57.1% vs. 27.7%, respectively), were more immunocompromised (61.5% vs. 22.8%, respectively), had more severe CDI (44.9% vs. 15.6%, respectively), experienced more renal failure (35.2% vs. 15.7%, respectively), and had more diagnoses by direct toxin detection (72.5% vs. 49.0%, respectively). Of the 91 participants, 13 had recurrence, resulting in a rCDI rate of 14.2%. There were no statistically significant differences between the group that experienced rCDI and the group that had no recurrence any of the eight variables examined. The authors concluded that BEZ was efficacious in preventing rCDI at a similar rate observed in MODIFY I/II, even in patients who were at a higher risk of rCDI. These findings should be supported by larger trials [50].

Immunocompromised patients are at an especially elevated risk of CDI [56]. Askar and colleagues published a retrospective cohort study to review BEZ use for the prevention of rCDI at 12 weeks, focusing on immunocompromised patients. The study was comprised of 53 participants, 23 in the BEZ group and 30 in the SoC group, in a large teaching facility. Consistent with the nature of the study, 82.6% in the BEZ group and 83.3% SoC group were immunocompromised (*p* = 0.9469). Inclusion criteria for the SoC group were those that had an initial CDI episode and received treatment as per institutional guidelines. Criteria for BEZ included one episode or more of CDI and immunocompromised status defined as solid organ transplant (SOT) or hematopoietic stem-cell transplant (HCT), receiving antineoplastic therapy, neutropenia (absolute neutrophil count ≤500 cells/microL), steroid use (prednisone equivalent of ≥20 mg/day), other immunosuppressants, or prior failed FMT, in addition to having 12 weeks of follow-up data. Bezlotoxumab was administered as a 10 mg/kg single IV infusion and SoC included VAN, MTZ or FDX for 10–14 days, depending on provider preference. The objective was to evaluate the real-life effectiveness and safety of BEZ in high-risk, immunocompromised participants. The primary end point was rCDI during 12 weeks of follow-up. Secondary end points were median time to an rCDI episode, rehospitalization rates, and patient safety. At week 12 after completion of SoC antibiotics, rCDI rates were 13% in the BEZ and 23.3% in the SoC group (*p* = 0.3464). Median time to recurrence was 45 days (range, 32 to 65) for the BEZ group and 29 days (range, 3 to 84) for the SoC group (*p* = 0.025). All-cause hospital readmissions were 22% in the BEZ group and 60% in the SoC group (*p* = 0.0057). CDI-attributable readmissions were 4% in the BEZ group and 10% in the SoC group (*p* = 0.4446). For the patient-safety end point, one patient experienced nausea and vomiting during BEZ infusion. The authors concluded that early real-life experience with BEZ appears promising and safe for reducing rCDI, particularly among immunocompromised and transplant patients, but these results must be interpreted with caution due to the small sample and should be further studied in larger trials [51].

Johnson and colleagues conducted a retrospective, matched-cohort study to investigate BEZ effectiveness in relation to rCDI and patient-specific risk factors in a real-world setting. Bezlotoxumab plus SoC (*n* = 54) was compared to SoC alone (*n* = 53) in participants aged ≥18 years who had received SoC (VAN or FDX), had one or more risk factors for rCDI, and documented follow-up 90 days after last dose of CDI therapy. Participants who received BEZ plus SoC were compared to historical controls receiving SoC alone in the two years immediately prior to BEZ use. Controls were matched 1:1 to the BEZ arm according to incidence of concurrent antibiotic use and number of prior CDI episodes. BEZ dosing was 10 mg/kg based on ABW, administered as a single intravenous dose. Treatment regimens with VAN and FDX were based on current practice guidelines. Risk factors for rCDI included age ≥ 65 years, immunocompromised status, prior episode of CDI, concomitant antibiotic use, proton-pump inhibitor use, severe CDI (Zar score ≥ 2), and proteinuria (urine total protein ≥ 30 mg/dL). The primary outcome was incidence of 90-day rCDI. Secondary outcomes were incidence of all-cause hospital readmission and all-cause mortality at 90 days, infusion-related reactions, and incidence of HF exacerbations. The incidence of rCDI at 90 days was 11% for the BEZ group and 43% in the SoC group (*p* < 0.001). All-cause hospital readmissions were 40% in the BEZ group and 64% in the SoC group (*p* = 0.011). There was no difference in all-cause mortality (BEZ 1.9%, SoC 0%; *p* = 0.999), HF exacerbation (BEZ 2.9%, SoC 7.1%; *p* = 0.503), or infusion-related reactions (BEZ 1.9%, SoC 0%, statistical analysis not performed) [52].

A separate study published by Johnson and colleagues was a single-center, retrospective analysis in 94 participants comparing rCDI at 90 days in SOT and HCT recipients receiving SoC monotherapy or BEZ in combination with SoC. BEZ treatment was based on an institutional protocol of 10 mg/kg of ABW, with a maximum dose of 1000 mg. SoC included oral VAN, FDX, or MTZ. The primary end point was incidence of rCDI at 90 days after completion of CDI antibiotics. Secondary outcomes included rCDI at 30 days, all-cause hospital readmission at 90 days, all-cause mortality at 90 days, incidence of BEZ infusion-related reactions, and HF exacerbations among participants with a preexisting diagnosis of HF. In an unadjusted analysis, no difference was observed in rates of rCDI between the BEZ and SoC groups (BEZ 16%, SoC 29%; *p* = 0.13). At 90 days, there was no difference in all-cause hospital readmission (BEZ 47%, SoC 50%; *p* = 0.67) or all-cause mortality (BEZ 0%, SoC 5%; *p* = 0.27). One patient experienced infusion-related nausea and vomiting that required cessation of BEZ treatment. There were no HF exacerbations (in those with baseline HF diagnosis) in the BEZ group and two in the SoC group (BEZ 0%, SoC 8%; *p* = 0.49). In a multivariate analysis of factors associated with 90-day rCDI, BEZ was associated with 75% lower odds than those who did not receive BEZ (OR, 0.28; 95% CI, 0.08 to 0.91; *p* = 0.03). The small sample and low incidence of safety events likely influenced the ability to detect a difference between the two groups [53].

Olmedo and colleagues conducted a small retrospective study of 16 patients in a large tertiary-care hospital describing the use and outcomes of CDI treated with BEZ. Participants were included in the study if they fulfilled the indications for the financing of BEZ and had three or more risk factors for rCDI. The primary objective was rCDI at 90 days in those participants treated with BEZ 10 mg/kg IV. Adverse events were also assessed. The follow-up period was 90 days or until the patient died. Two participants died before the 90-day follow-up period. Of the remaining 14 participants, three had a recurrence, resulting in a rCDI rate of 21.4%. No significant ADRs were reported. The results of this trial generally align with results from other trials and support the efficacy of BEZ for patients with multiple risk factors for rCDI, but the very small sample prevents any definitive conclusions [54].

## 6. Discussion and Conclusions

*C. difficile* infections, and particularly rCDIs, cause a significant strain on the healthcare system. One of the biggest challenges with the treatment of CDIs is that, even with pharmacotherapy treatment, there is still a high incidence of rCDIs. Use of MTZ, a traditional treatment for CDI, is no longer recommended for use except in limited circumstances because the risk of rCDIs is higher than with VAN [57,58]. Further, the risk of rCDIs is higher with VAN compared to FDX, which is why FDX is now the preferred treatment option [59,60,61,62]. However, use of FDX does not negate the risk of rCDIs. As such, effective pharmacotherapy options to reduce the risk of rCDIs are needed. BEZ represents a powerful tool in the armamentarium against CDI. Most published data with BEZ demonstrate its’ effectiveness at preventing rCDI. Furthermore, it is associated with few adverse effects. Additionally, BEZ is effective at preventing rCDI when used in primary CDI cases and effective in preventing further recurrences in someone already experiencing rCDI. Studies support the efficacy of BEZ in otherwise healthy patients, but many studies show that BEZ may be most beneficial in patients with risk factors for rCDI.

The 2021 focused CDI guideline update recommends the use of BEZ for anyone experiencing a recurrent infection within six months of a previous infection, and also recommends use in a primary CDI episode if the patient has at least one risk factor for rCDI (specifically, age ≥65 years, immunosuppression, and severe CDI upon presentation) if logistical concerns are not an issue, such as cost and feasibility of IV therapy [11]. Literature published since the 2021 focused update was released supports the guideline recommendation. In particular, real-world data support the conclusions observed in larger clinical trials. At present, the literature is lacking in support for the use of BEZ outside of what is currently recommended by the guidelines. Some studies have shown promise for the use of BEZ in particular settings, such as immunosuppression, but the small samples prevent definitive conclusions, and larger trials are needed in the future.

Published literature has also demonstrated that BEZ is well tolerated overall, with infusion-related reactions being the most common. However, HF exacerbations can occur with BEZ therapy, so use in these patients should be avoided unless the benefits clearly outweigh the risks. Finally, as previously mentioned, logistical concerns may limit BEZ use in real-world settings. For example, in outpatients being treated for CDI, lack of access to an infusion center may be problematic. Additionally, cost may prohibit the use in some patients, with the average wholesale cost of BEZ being $4560 per 1000 mg vial [63]. There are indirect costs associated with BEZ therapy, such as obtaining IV access and administration time. However, two studies have supported the cost-effectiveness of BEZ therapy [64,65]. Both trials examined cost-effectiveness of BEZ using the same risk factors for rCDI used in the MODIFY trials. One trial used European cost estimates and found that the cost effectiveness of BEZ was highest for patients ≥65 years who had experienced at least one rCDI in the preceding 6 months, followed by those of any age who had experienced at least one rCDI in the preceding 6 months, followed by patents ≥65 years [64]. The other trial used United States cost estimates and found similar results, with BEZ being most cost-effective in patients ≥65 years and those who were immunocompromised [65]. Overall, BEZ appears to be cost-effective, especially for patients at high risk of rCDI, but this is likely to vary between patients based on insurance coverage. Clinicians should strongly consider the use of BEZ in patients with a risk factor for rCDI and those experiencing rCDI if logistical issues, such as cost and administration, can be overcome.

## Figures and Tables

**Table 1:** Comparison of the IDSA/SHEA and ACG clinical practice guidelines.

**American College of Gastroenterology Guidelines**	
2013	Not addressed since bezlotoxumab was not available
2021	Bezlotoxumab should be considered for prevention of CDI recurrence in patients who are at high risk of recurrence. * (Conditional recommendation, moderate quality of evidence)
Rationale	MODIFY trials found a number needed to treat (NNT) of 10 to prevent one episode of rCDI with BEZ. NNT to prevent one rCDI was 6 for patients >65 years and for those with >1 CDI episode within the past 6 months.
**Infectious Diseases Society of America/Society for Healthcare Epidemiology of America Guidelines**	
2017	These guidelines note the availability of BEZ, but specific recommendations were not made because the guidelines had been completed by the time of BEZ approval.
2021	BEZ can be used as a cointervention with standard-of-care antibiotics rather than antibiotics alone for patients with rCDI within the last 6 months (conditional recommendation, very low certainty of evidence). Implementation may be limited by feasibility concerns (i.e., cost, need for intravenous access). If logistics/feasibility is not an issue, BEZ can be considered for patients with a primary CDI episode and other risk factors for rCDI.
Rationale	Post hoc analysis of MODIFY trials demonstrated the addition of BEZ reduced rCDI after initial clinical cure at 12 weeks. Real-world studies found similar reductions in rCDI after initial clinical cure among patients at high risk of rCDI who received BEZ.

* High risk of recurrence: age > 65 years and one of the following risk factors: experiencing their second episode of CDI within the past 6 months, immunocompromised, or severe CDI.

**Table 2:** Rates of rCDI in patients with pre-specified risk factors [34].

Risk Factor	BEZ, %	Placebo, %	Difference, % [95% CI]
Age > 65 years	19.3	39.4	−20.1 [−27.0 to −13.2]
History of CDI	31.6	49.5	−17.9 [−27.7 to −7.6]
Immunocompromised	19.0	36.0	−17.0 [−28.0 to −6.0]
Severe CDI	15.9	31.5	−15.6 [−28.0 to −2.8]
Ribotype 027/078/244	28.2	41.1	−12.9 [−26.8 to 1.6]

**Table 3:** Rates of rCDI according to number of prespecified risk factors [34].

NO. OF RISK FACTORS	BEZ, %	PLACEBO, %	DIFFERENCE, % [95% CI]
0	18.8	20.9	−2.1 [−11.1 to 6.9]
≥1	21.2	37.2	−15.9 [−21.6 to −10.2]
1	17.1	31.3	−14.2 [−21.9 to −6.4]
2	26.9	41.1	−14.2 [−24.0 to −4.1
≥3	21.2	46.1	−24.8 [−39.1 to −9.3]

**Table 4:** Highlights of Real-World Trials with Bezlotoxumab [49,50,51,52,53,54].

Reference	Design	Regimen	Number of Patients	Patient Demographics (%)				*C. diff* Recurrence Rate (%)	Statistics
Escudero-Sanchez et al. [49]	Matched cohort study of FDX monotherapy vs. SoC + BEZ			-	≥65 YOA	Immunosuppressed	Severe *C. diff*		OR 1.45 95% CI 0.71–2.96 *p* = 0.29
		FDX 200 mg BID	244	FDX	60.7	38.9	40.2	19.3	
		BEZ 10 mg/kg IV + SoC (VAN +/− MTZ)	78	BEZ + SoC	64.1	66.7	38.5	14.1	
Escudero-Sanchez et al. [50]	Retrospective, multi-center, cohort study to examine eight risk factors for rCDI	BEZ 10 mg/kg IV + SoC	91	%	≥65 YOA	IS	Severe *C. diff*	14.2	Risk factors assessed individually
				Recurrence	61.5	53.9	53.9		
				No recurrence	68	62.8	43.6		
Askar et al. [51]	Retrospective cohort of SoC +/− BEZ at 12 weeks			%	≥60 YOA	IS	Severe *C. diff*		*p* = 0.3464
		SoC alone (VAN, MTZ, or FDX) for 10–14 days	30	SoC	43.3	83.3	-	23.3	
		SoC + BEZ 10 mg/kg IV	23	SoC + BEZ	52.2	82.6	-	13.04	
Johnson et al. [52]	Matched, retrospective cohort study of SoC +/− BEZ at 90 days			%	≥65 YOA	IS	Severe *C. diff*		ARR 32.1 95% CI 16.2–47.9 *p* ≤ 0.001
		SoC = VAN or FDX	53	SoC	28	49	34	43	
		BEZ 10 mg/kg IV + SoC	53	BEZ	38	77	23	11	
Johnson et al. [53]	Single-center retrospective analysis of SoC +/− BEZ in transplant patients			%	≥65 YOA	IS	Severe *C. diff*		*p* = 0.13
		SoC = VAN, MTZ, or FDX	56	SoC	21	100	32	29	
		SoC + BEZ 10 mg/kg IV	38	SoC + BEZ	26	100	13	16	
Olmedo et al. [54]	Retrospective study to describe outcomes in patients treated with BEZ at high risk of rCDI	BEZ 10 mg/kg IV	16	%	≥65 YOA	IS	Severe *C. diff*	21.4 *	Not provided
				BEZ	69	87.5	56		

ARR = absolute risk reduction; BEZ = bezlotoxumab; FDX = fidaxomicin; IS = immunosuppressed; MTZ = metronidazole; rCDI = recurrent *C. difficile* infection; SoC = standard of care; VAN = vancomycin; YOA = years of age. * Represents 3/14 patients due to death of 2 patients.

## Data Availability

Not applicable.
